# LiCe_9_Mo_16_O_35_

**DOI:** 10.1107/S160053681200801X

**Published:** 2012-02-29

**Authors:** Patrick Gougeon, Philippe Gall

**Affiliations:** a Sciences Chimiques de Rennes, UMR CNRS No. 6226, Université de Rennes I - INSA Rennes, Avenue du Général Leclerc, 35042 Rennes CEDEX, France

## Abstract

The structure of lithium nona­cerium hexa­deca­molybdenum penta­trideca­oxide, LiCe_9_Mo_16_O_35_, is isotypic with LiNd_9_Mo_16_O_35_ [Gougeon Gall, Cuny, Gautier, Le Polles, Delevoye & Trebosc (2011 ▶). *Chem. Eur. J.* **17**, 13806–13813]. It is characterized by Mo_16_O_26_^*i*^O_10_^*a*^ units (where *i* = inner and *a* = apical) containing Mo_16_ clusters that share some of their O atoms to form infinite molybdenum cluster chains running parallel to the *b* axis and separated by Li^+^ and Ce^3+^ cations. The Mo_16_ cluster units are centred at Wyckoff positions 2*c* and have point-group symmetry 2/*m*. The Li^+^ atom, in a flattened octa­hedron of O atoms, is in a 2*a* Wyckoff position with 2/*m* symmetry. The Ce^3+^ cations have coordination numbers to the O atoms of 6, 9 or 10. Two Ce, two Mo and five O atoms lie on sites with *m* symmetry (Wyckoff site 4*i*), and one Ce and one O atom on sites with 2/*m* symmetry (Wyckoff sites 2*b* and 2*d*, respectively).

## Related literature

For the crystal structure of the LiNd_9_Mo_16_O_35_ compound, see: Gougeon *et al.* (2011 ▶). For details of the *i*- and *a*-type ligand notation, see: Schäfer & von Schnering (1964 ▶). For compounds containing Mo_10_ clusters, see: Hibble *et al.* (1988 ▶); Dronskowski & Simon (1989 ▶); Gougeon *et al.* (1990 ▶, 1991 ▶, 2003 ▶, 2007 ▶); Dronskowski *et al.* (1991 ▶); Gall *et al.* (1993 ▶, 1995 ▶, 1999 ▶); Gall & Gougeon (1993 ▶, 1994*a* ▶,*b* ▶, 1998 ▶); Gougeon & Gall (2002 ▶). The oxidation states of the Mo, Ce and Li atoms were estimated using the data given by Brown & Wu (1976 ▶).

## Experimental

### 

#### Crystal data

LiCe_9_Mo_16_O_35_
*M* *_r_* = 3363.06
Monoclinic, 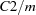

*a* = 18.3000 (2) Å
*b* = 8.6326 (1) Å
*c* = 9.8172 (1) Å
β = 102.0953 (7)°
*V* = 1516.46 (3) Å^3^
*Z* = 2
Mo *K*α radiation
μ = 19.66 mm^−1^
*T* = 293 K
0.15 × 0.12 × 0.06 mm

#### Data collection

Nonius KappaCCD diffractometer
Absorption correction: multi-scan (*PLATON*; Spek, 2009 ▶) *T*_min_ = 0.190, *T*_max_ = 0.418
21270 measured reflections
3511 independent reflections
3383 reflections with *I* > 2σ(*I*)
*R*_int_ = 0.027

#### Refinement

*R*[*F* ^2^ > 2σ(*F* ^2^)] = 0.016
*wR*(*F* ^2^) = 0.033
*S* = 1.29
3511 reflections
158 parameters
Δρ_max_ = 1.13 e Å^−3^
Δρ_min_ = −1.33 e Å^−3^

### 

Data collection: *COLLECT* (Nonius, 1998 ▶); cell refinement: *COLLECT*; data reduction: *EVALCCD* (Duisenberg *et al.*, 2003 ▶); program(s) used to solve structure: *SIR97* (Altomare *et al.*, 1999 ▶); program(s) used to refine structure: *SHELXL97* (Sheldrick, 2008 ▶); molecular graphics: *DIAMOND* (Bergerhoff, 1996 ▶); software used to prepare material for publication: *SHELXL97*.

## Supplementary Material

Crystal structure: contains datablock(s) I, global. DOI: 10.1107/S160053681200801X/ru2029sup1.cif

Structure factors: contains datablock(s) I. DOI: 10.1107/S160053681200801X/ru2029Isup2.hkl

Additional supplementary materials:  crystallographic information; 3D view; checkCIF report

## Figures and Tables

**Table 1:** Selected bond lengths (Å)

Li—O7	1.861 (3)
Li—O10	2.4135 (16)
Ce1—O3^i^	2.3603 (16)
Ce1—O12	2.3916 (16)
Ce1—O1^ii^	2.4055 (5)
Ce1—O8	2.4159 (16)
Ce1—O10^iii^	2.5499 (19)
Ce1—O4^ii^	2.7161 (18)
Ce1—O2^ii^	2.874 (2)
Ce1—O11	2.8865 (16)
Ce1—O7^iv^	2.8959 (17)
Ce2—O12	2.337 (3)
Ce2—O3^v^	2.3829 (19)
Ce2—O3	2.3829 (19)
Ce2—O1^iv^	2.622 (2)
Ce2—O2^vi^	2.6352 (17)
Ce2—O2^iv^	2.6352 (17)
Ce2—O5^ii^	2.8054 (16)
Ce2—O5^vii^	2.8054 (16)
Ce2—O2^vii^	2.9625 (17)
Ce2—O2^ii^	2.9625 (17)
Ce3—O8	2.3670 (17)
Ce3—O8^viii^	2.3670 (17)
Ce3—O4^ii^	2.5309 (18)
Ce3—O4^ix^	2.5309 (18)
Ce3—O5^viii^	2.7980 (17)
Ce3—O5	2.7980 (17)
Ce3—O4^viii^	3.0001 (17)
Ce3—O4	3.0001 (17)
Ce3—O11^ii^	3.089 (3)
Ce4—O6^x^	2.280 (2)
Ce4—O6	2.280 (2)
Ce4—O3	2.4244 (19)
Ce4—O3^x^	2.4244 (19)
Ce4—O3^v^	2.4244 (19)
Ce4—O3^xi^	2.4244 (19)
Mo1—O2	1.9759 (16)
Mo1—O2^v^	1.9759 (16)
Mo1—O4^v^	2.0332 (17)
Mo1—O4	2.0332 (17)
Mo1—O1	2.045 (3)
Mo1—Mo3	2.7003 (3)
Mo1—Mo2	2.7129 (3)
Mo2—O3^ix^	2.0277 (16)
Mo2—O2	2.077 (2)
Mo2—O7	2.0773 (16)
Mo2—O5	2.0985 (16)
Mo2—O10	2.0997 (19)
Mo2—Mo4^ii^	2.6412 (3)
Mo2—Mo5^xii^	2.7691 (3)
Mo2—Mo3	2.7739 (2)
Mo2—Mo2^v^	2.7851 (4)
Mo3—O8	2.0315 (16)
Mo3—O5	2.0688 (16)
Mo3—O11	2.0795 (16)
Mo3—O4	2.0860 (19)
Mo3—Mo3^v^	2.7338 (4)
Mo3—Mo4^ii^	2.7375 (3)
Mo3—Mo5	2.7405 (3)
Mo3—Mo4^xiii^	2.8391 (3)
Mo3—Mo5^xii^	2.8928 (3)
Mo4—O6	2.0145 (14)
Mo4—O8^xiv^	2.0758 (19)
Mo4—O5^ii^	2.0797 (19)
Mo4—O10^ii^	2.0857 (16)
Mo4—O9	2.1141 (2)
Mo4—Mo4^xv^	2.7958 (3)
Mo5—O11	2.078 (3)
Mo5—O10^xii^	2.0950 (16)
Mo5—O10^iii^	2.0950 (16)
Mo5—Mo5^xii^	2.9030 (5)

Symmetry codes: (i) 
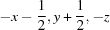
; (ii) 
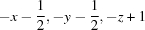
; (iii) 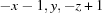; (iv) 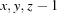; (v) 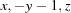; (vi) 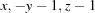; (vii) 
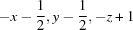
; (viii) 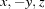; (ix) 
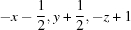
; (x) 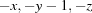; (xi) 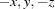; (xii) 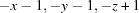; (xiii) 
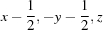
; (xiv) 
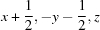
; (xv) 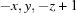.

**Fig. 1.**
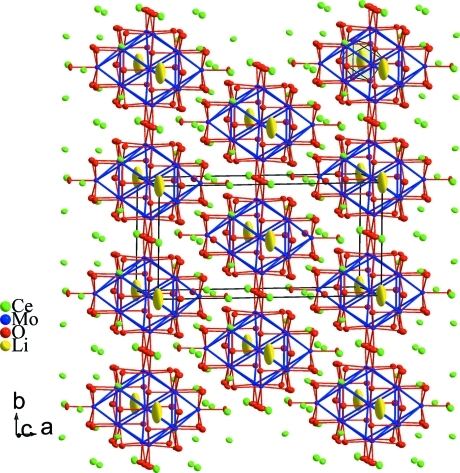
View of the crystal structure of LiCe9Mo16O35 (Displacement ellipsoids are drawn at the 97% probability level).

**Fig. 2.**
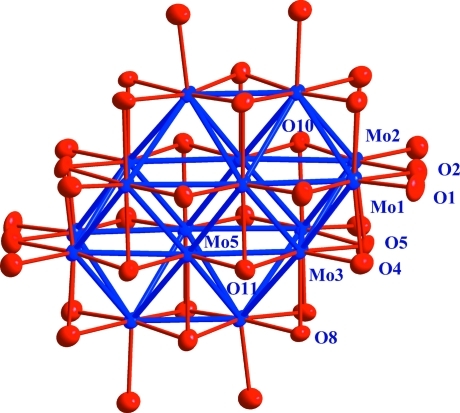
Plot showing the atom-numbering scheme of the Mo16O26iO10a units. Displacement ellipsoids are drawn at the 97% probability level.

## supplementary crystallographic information

### Comment

LiCe_9_Mo_16_O_35_ is isotopic with the LiNd_9_Mo_16_O_35_ (Gougeon *et al.*, 2011) structure type. The crystal structure (Fig. 1) is based on Mo_16_O_26_^i^O_10_^a^ cluster units (Fig. 2) sharing two O^i^ or four O^a^ ligands (for details of the i- and a-type ligand notation, see Schäfer & von Schnering (1964)) to form infinite molybdenum cluster chains running parallel to the b axis. The Mo_16_ core of the Mo_16_O_26_^i^O_10_^a^ unit can be seen as resulting of the fusion of two bioctahedral Mo_10_ clusters through the sharing of three edges per Mo_10_ cluster. The Mo_10_ cluster results itself from metal edge-sharing of two Mo_6_ octahedra and was first observed forming infinite chains in the MMo_5_O_8_ (M = Ca, Sr, Sn, Pb, La–Gd) compounds (Hibble *et al.*, 1988; Dronskowski & Simon, 1989; Gougeon *et al.*, 1990, 1991, 2003, 2007; Dronskowski *et al.*, 1991; Gall *et al.*, 1993, 1995; Gall & Gougeon, 1994*a*,*b*; Gougeon & Gall, 2002) and more later as isolated cluster in the R_16_Mo_21_O_56_ (R = La, Ce, Pr, and Nd) series (Gall & Gougeon, 1993, 1998; Gall *et al.*, 1999). The Mo_16_O_26_^i^O_10_^a^ cluster is centered on 2b position and thus has point-group symmetry 2/m. The Mo—Mo distances lie between 2.6412 (3) and 2.9030 (5) Å compared to 2.725 Å in the Mo metal and the Mo—O distances between 1.9759 (16) and 2.11407 (18) Å. The Mo—O chains, which have the connectivity formula Mo_16_O_24_^i^O_2/2_^i-i^O_6_^a^O_4/2_^a-a^ are separated by the Li^+^ and Ce^3+^ cations. The Li^+^ cation occupies a highly tetragonally distorted octahedral site of O atoms of symmetry 2/m centered at the origin of the unit cell. The Li—O distances in the equatorial plane are 2.4135 (16) Å [Li—O10] and the two trans Li—O7 bonds are 1.861 (3) Å. The coordination numbers of the Ce ions are 6, 9 or 10 with Ce—O distances spreading over a wide range [2.280 (2) to 3.089 (3) Å]. By using the bond-length–bond-strength formula (Brown & Wu, 1976) for the Mo—O, Ce—O and Li—O bonds (s = [d(Mo—O)/1.882]^-6^, s = [d(Ce—O)/2.160]^-6.5^ and s = [d(Li—O)/1.378]^-4.065^), an assignment of oxidation states to the Mo, Ce and Li atoms was made. The valence of each independent Mo atom was determined as follows: Mo(1) +3.36, Mo(2) +2.78, Mo(3) +2.29, Mo(4) + 2.81, and Mo(5) + 1.6. From these values, we could deduce an average Mo oxidation state of + 2.60 which is close to that based on the stoichiometry, + 2.625, when considering all the Ce ions as trivalent and the Li ion monovalent. Bond-valence sums of the Ce—O bonds was +3.08, +3.11, +3.09, and +3.30 for Ce1, Ce2, Ce3, and Ce4, respectively. For the Li atom, a value of +1.00 was found. It is interesting to note that for the total valence sum Σ(Mo—O) + Σ(Ce—O) + Σ(Li—O), we obtained a value of 70.5 per formula unit, which is in very good agreement with the theoretical value of 70 based on the 35 O atoms.

### Experimental

Single crystals of LiCe_9_Mo_16_O_35_ were obtained from a mixture of Li_2_MoO_4_, CeO_2_, MoO_3_, and Mo with the nominal composition Li_2_CeMo_6_O_12_. Before use, Mo powder was reduced under H_2_ flowing gas at 1273 K during 10 h in order to eliminate any trace of oxygen. The initial mixture (ca 4 g) was cold pressed and loaded into a molybdenum crucible, which was sealed under a low argon pressure using an arc welding system. The charge was heated at the rate of 300 K/h up to 2000 K, temperature which was held for 18 h, then cooled at 100 K/h down to 1373 K and finally furnace cooled.

### Crystal data

**Table:**

LiCe_9_Mo_16_O_35_	*F*(000) = 2954
*M**_r_* = 3363.06	*D*_x_ = 7.365 Mg m^−^^3^
Monoclinic, *C*2/*m*	Mo *K*α radiation, λ = 0.71069 Å
*a* = 18.3000 (2) Å	Cell parameters from 21270 reflections
*b* = 8.6326 (1) Å	θ = 0.9–35.0°
*c* = 9.8172 (1) Å	µ = 19.66 mm^−^^1^
β = 102.0953 (7)°	*T* = 293 K
*V* = 1516.46 (3) Å^3^	Multi-faceted fragment, black
*Z* = 2	0.15 × 0.12 × 0.06 mm

### Data collection

**Table:**

Nonius KappaCCD diffractometer	3511 independent reflections
Radiation source: fine-focus sealed tube	3383 reflections with *I* > 2σ(*I*)
Graphite monochromator	*R*_int_ = 0.027
φ scans (κ = 0) and additional ω scans	θ_max_ = 35.0°, θ_min_ = 3.5°
Absorption correction: multi-scan (*PLATON*; Spek, 2009)	*h* = −26→29
*T*_min_ = 0.190, *T*_max_ = 0.418	*k* = −13→13
21270 measured reflections	*l* = −15→10

### Refinement

**Table:**

Refinement on *F*^2^	Primary atom site location: structure-invariant direct methods
Least-squares matrix: full	Secondary atom site location: difference Fourier map
*R*[*F*^2^ > 2σ(*F*^2^)] = 0.016	*w* = 1/[σ^2^(*F*_o_^2^) + (0.*P*)^2^ + 6.2844*P*] where *P* = (*F*_o_^2^ + 2*F*_c_^2^)/3
*wR*(*F*^2^) = 0.033	(Δ/σ)_max_ = 0.004
*S* = 1.29	Δρ_max_ = 1.13 e Å^−^^3^
3511 reflections	Δρ_min_ = −1.33 e Å^−^^3^
158 parameters	Extinction correction: *SHELXL97* (Sheldrick, 2008), Fc^*^=kFc[1+0.001xFc^2^λ^3^/sin(2θ)]^-1/4^
0 restraints	Extinction coefficient: 0.00166 (3)

### Special details

**Table:**

Geometry. All esds (except the esd in the dihedral angle between two l.s. planes) are estimated using the full covariance matrix. The cell esds are taken into account individually in the estimation of esds in distances, angles and torsion angles; correlations between esds in cell parameters are only used when they are defined by crystal symmetry. An approximate (isotropic) treatment of cell esds is used for estimating esds involving l.s. planes.
Refinement. Refinement of F^2^ against ALL reflections. The weighted R-factor wR and goodness of fit S are based on F^2^, conventional R-factors R are based on F, with F set to zero for negative F^2^. The threshold expression of F^2^ > 2sigma(F^2^) is used only for calculating R-factors(gt) etc. and is not relevant to the choice of reflections for refinement. R-factors based on F^2^ are statistically about twice as large as those based on F, and R-factors based on ALL data will be even larger.

### Fractional atomic coordinates and isotropic or equivalent isotropic displacement parameters (Å^2^)

**Table:**

	*x*	*y*	*z*	*U*_iso_*/*U*_eq_	
Li	−0.5000	−0.5000	1.0000	0.038 (4)	
Ce1	−0.345982 (8)	−0.273486 (15)	0.201055 (12)	0.00556 (3)	
Ce2	−0.191529 (11)	−0.5000	0.071345 (17)	0.00625 (4)	
Ce3	−0.298077 (11)	0.0000	0.534154 (17)	0.00582 (4)	
Ce4	0.0000	−0.5000	0.0000	0.00664 (5)	
Mo1	−0.290730 (16)	−0.5000	0.74596 (2)	0.00368 (5)	
Mo2	−0.401044 (12)	−0.33869 (2)	0.828637 (17)	0.00338 (4)	
Mo3	−0.388827 (11)	−0.34166 (2)	0.551746 (17)	0.00319 (4)	
Mo4	−0.000772 (11)	−0.31629 (2)	0.357312 (17)	0.00311 (4)	
Mo5	−0.490663 (16)	−0.5000	0.35678 (2)	0.00316 (5)	
O1	−0.17701 (15)	−0.5000	0.8112 (2)	0.0070 (4)	
O2	−0.28523 (11)	−0.3349 (2)	0.88711 (16)	0.0064 (3)	
O3	−0.10014 (11)	−0.6759 (2)	0.02198 (16)	0.0062 (3)	
O4	−0.27246 (11)	−0.3355 (2)	0.60863 (16)	0.0059 (3)	
O5	−0.38352 (11)	−0.1616 (2)	0.69257 (16)	0.0051 (3)	
O6	0.00695 (16)	−0.5000	0.2346 (2)	0.0066 (4)	
O7	−0.40015 (16)	−0.5000	0.9860 (2)	0.0067 (4)	
O8	−0.38482 (10)	−0.1769 (2)	0.40574 (15)	0.0050 (3)	
O9	0.0000	−0.5000	0.5000	0.0059 (6)	
O10	−0.51815 (10)	−0.33622 (19)	0.79643 (16)	0.0050 (3)	
O11	−0.37455 (15)	−0.5000	0.3998 (2)	0.0052 (4)	
O12	−0.26903 (15)	−0.5000	0.2327 (2)	0.0063 (4)	

### Atomic displacement parameters (Å^2^)

**Table:**

	*U*^11^	*U*^22^	*U*^33^	*U*^12^	*U*^13^	*U*^23^
Li	0.007 (6)	0.070 (12)	0.037 (7)	0.000	0.005 (5)	0.000
Ce1	0.00563 (6)	0.00466 (5)	0.00630 (5)	0.00095 (4)	0.00106 (4)	0.00077 (3)
Ce2	0.00587 (9)	0.00703 (8)	0.00639 (6)	0.000	0.00251 (5)	0.000
Ce3	0.00568 (9)	0.00564 (7)	0.00553 (6)	0.000	−0.00025 (5)	0.000
Ce4	0.00575 (12)	0.00932 (11)	0.00530 (9)	0.000	0.00216 (7)	0.000
Mo1	0.00269 (12)	0.00349 (10)	0.00447 (9)	0.000	−0.00012 (7)	0.000
Mo2	0.00339 (9)	0.00276 (7)	0.00372 (6)	0.00000 (6)	0.00011 (5)	−0.00036 (5)
Mo3	0.00291 (9)	0.00265 (7)	0.00385 (6)	−0.00007 (6)	0.00036 (5)	0.00014 (5)
Mo4	0.00306 (9)	0.00221 (7)	0.00386 (6)	−0.00005 (6)	0.00029 (5)	−0.00010 (5)
Mo5	0.00309 (12)	0.00254 (10)	0.00372 (9)	0.000	0.00047 (7)	0.000
O1	0.0034 (11)	0.0067 (10)	0.0095 (9)	0.000	−0.0014 (7)	0.000
O2	0.0061 (8)	0.0056 (7)	0.0068 (6)	0.0004 (6)	−0.0004 (5)	−0.0021 (5)
O3	0.0073 (8)	0.0050 (7)	0.0063 (6)	0.0000 (6)	0.0010 (5)	0.0013 (5)
O4	0.0038 (8)	0.0062 (7)	0.0077 (6)	−0.0006 (6)	0.0010 (5)	0.0006 (5)
O5	0.0044 (8)	0.0043 (7)	0.0062 (6)	−0.0001 (6)	0.0006 (5)	0.0000 (5)
O6	0.0110 (12)	0.0039 (10)	0.0047 (8)	0.000	0.0013 (7)	0.000
O7	0.0089 (12)	0.0051 (10)	0.0058 (8)	0.000	0.0011 (7)	0.000
O8	0.0039 (8)	0.0051 (7)	0.0055 (6)	−0.0001 (6)	0.0000 (5)	0.0009 (5)
O9	0.0100 (17)	0.0036 (13)	0.0041 (11)	0.000	0.0014 (10)	0.000
O10	0.0037 (8)	0.0041 (7)	0.0073 (6)	−0.0001 (6)	0.0013 (5)	0.0000 (5)
O11	0.0046 (11)	0.0060 (10)	0.0053 (8)	0.000	0.0017 (7)	0.000
O12	0.0063 (12)	0.0063 (10)	0.0060 (8)	0.000	0.0002 (7)	0.000

### Geometric parameters (Å, º)

**Table:**

Li—O7^i^	1.861 (3)	Mo3—O11	2.0795 (16)
Li—O7	1.861 (3)	Mo3—O4	2.0860 (19)
Li—O10^ii^	2.4135 (16)	Mo3—Mo3^ii^	2.7338 (4)
Li—O10^iii^	2.4135 (16)	Mo3—Mo4^v^	2.7375 (3)
Li—O10^i^	2.4135 (16)	Mo3—Mo5	2.7405 (3)
Li—O10	2.4135 (16)	Mo3—Mo4^xv^	2.8391 (3)
Ce1—O3^iv^	2.3603 (16)	Mo3—Mo5^xiv^	2.8928 (3)
Ce1—O12	2.3916 (16)	Mo4—O6	2.0145 (14)
Ce1—O1^v^	2.4055 (5)	Mo4—O8^xvi^	2.0758 (19)
Ce1—O8	2.4159 (16)	Mo4—O5^v^	2.0797 (19)
Ce1—O10^vi^	2.5499 (19)	Mo4—O10^v^	2.0857 (16)
Ce1—O4^v^	2.7161 (18)	Mo4—O9	2.1141 (2)
Ce1—O2^v^	2.874 (2)	Mo4—Mo2^v^	2.6412 (3)
Ce1—O11	2.8865 (16)	Mo4—Mo5^xvii^	2.7367 (2)
Ce1—O7^vii^	2.8959 (17)	Mo4—Mo3^v^	2.7375 (3)
Ce2—O12	2.337 (3)	Mo4—Mo4^xviii^	2.7958 (3)
Ce2—O3^ii^	2.3829 (19)	Mo4—Mo3^xvi^	2.8391 (3)
Ce2—O3	2.3829 (19)	Mo4—Mo4^ii^	3.1718 (4)
Ce2—O1^vii^	2.622 (2)	Mo5—O11	2.078 (3)
Ce2—O2^viii^	2.6352 (17)	Mo5—O10^xiv^	2.0950 (16)
Ce2—O2^vii^	2.6352 (17)	Mo5—O10^vi^	2.0950 (16)
Ce2—O5^v^	2.8054 (16)	Mo5—Mo4^xix^	2.7367 (2)
Ce2—O5^ix^	2.8054 (16)	Mo5—Mo4^xv^	2.7367 (2)
Ce2—O2^ix^	2.9625 (17)	Mo5—Mo3^ii^	2.7405 (3)
Ce2—O2^v^	2.9625 (17)	Mo5—Mo2^xiv^	2.7691 (3)
Ce3—O12^v^	2.360 (2)	Mo5—Mo2^vi^	2.7691 (3)
Ce3—O8	2.3670 (17)	Mo5—Mo3^xiv^	2.8928 (3)
Ce3—O8^x^	2.3670 (17)	Mo5—Mo3^vi^	2.8928 (3)
Ce3—O4^v^	2.5309 (18)	Mo5—Mo5^xiv^	2.9030 (5)
Ce3—O4^xi^	2.5309 (18)	O1—Ce1^v^	2.4055 (5)
Ce3—O5^x^	2.7980 (17)	O1—Ce1^ix^	2.4055 (5)
Ce3—O5	2.7980 (17)	O1—Ce2^xx^	2.622 (2)
Ce3—O4^x^	3.0001 (17)	O1—Ce3^v^	3.325 (2)
Ce3—O4	3.0001 (17)	O2—Ce2^xx^	2.6352 (17)
Ce3—O11^v^	3.089 (3)	O2—Ce1^v^	2.874 (2)
Ce4—O6^xii^	2.280 (2)	O2—Ce2^v^	2.9625 (17)
Ce4—O6	2.280 (2)	O3—Mo2^ix^	2.0277 (16)
Ce4—O3	2.4244 (19)	O3—Ce1^xxi^	2.3603 (16)
Ce4—O3^xii^	2.4244 (19)	O4—Ce3^v^	2.5309 (18)
Ce4—O3^ii^	2.4244 (19)	O4—Ce1^v^	2.7161 (18)
Ce4—O3^xiii^	2.4244 (19)	O5—Mo4^v^	2.0797 (19)
Mo1—O2	1.9759 (16)	O5—Ce2^v^	2.8054 (16)
Mo1—O2^ii^	1.9759 (16)	O6—Mo4^ii^	2.0145 (14)
Mo1—O4^ii^	2.0332 (17)	O7—Mo2^ii^	2.0773 (16)
Mo1—O4	2.0332 (17)	O7—Ce1^xx^	2.8959 (17)
Mo1—O1	2.045 (3)	O7—Ce1^xxii^	2.8959 (17)
Mo1—Mo3^ii^	2.7003 (3)	O8—Mo4^xv^	2.0758 (19)
Mo1—Mo3	2.7003 (3)	O9—Mo4^ii^	2.1141 (2)
Mo1—Mo2^ii^	2.7129 (3)	O9—Mo4^xviii^	2.1141 (2)
Mo1—Mo2	2.7129 (3)	O9—Mo4^xxiii^	2.1141 (2)
Mo2—O3^xi^	2.0277 (16)	O9—Ce3^v^	3.6397 (2)
Mo2—O2	2.077 (2)	O9—Ce3^xxiv^	3.6397 (2)
Mo2—O7	2.0773 (16)	O10—Mo4^v^	2.0857 (16)
Mo2—O5	2.0985 (16)	O10—Mo5^xiv^	2.0950 (16)
Mo2—O10	2.0997 (19)	O10—Ce1^vi^	2.5499 (19)
Mo2—Mo4^v^	2.6412 (3)	O11—Mo3^ii^	2.0795 (16)
Mo2—Mo5^xiv^	2.7691 (3)	O11—Ce1^ii^	2.8865 (16)
Mo2—Mo3	2.7739 (2)	O11—Ce3^v^	3.089 (3)
Mo2—Mo2^ii^	2.7851 (4)	O12—Ce3^v^	2.360 (2)
Mo3—O8	2.0315 (16)	O12—Ce1^ii^	2.3916 (16)
Mo3—O5	2.0688 (16)		
			
O7^i^—Li—O7	180.0	O2^ii^—Mo1—Mo2	95.29 (6)
O7^i^—Li—O10^ii^	95.41 (7)	O4^ii^—Mo1—Mo2	142.46 (6)
O7—Li—O10^ii^	84.59 (7)	O4—Mo1—Mo2	94.36 (5)
O7^i^—Li—O10^iii^	84.59 (7)	O1—Mo1—Mo2	132.72 (5)
O7—Li—O10^iii^	95.41 (7)	Mo3^ii^—Mo1—Mo2	92.568 (10)
O10^ii^—Li—O10^iii^	180.0	Mo3—Mo1—Mo2	61.651 (7)
O7^i^—Li—O10^i^	84.59 (7)	Mo2^ii^—Mo1—Mo2	61.767 (10)
O7—Li—O10^i^	95.41 (7)	O2—Mo1—Ce2^xx^	52.36 (5)
O10^ii^—Li—O10^i^	108.28 (8)	O2^ii^—Mo1—Ce2^xx^	52.36 (5)
O10^iii^—Li—O10^i^	71.72 (8)	O4^ii^—Mo1—Ce2^xx^	120.27 (5)
O7^i^—Li—O10	95.41 (7)	O4—Mo1—Ce2^xx^	120.27 (5)
O7—Li—O10	84.59 (7)	O1—Mo1—Ce2^xx^	52.01 (7)
O10^ii^—Li—O10	71.72 (8)	Mo3^ii^—Mo1—Ce2^xx^	145.707 (6)
O10^iii^—Li—O10	108.28 (8)	Mo3—Mo1—Ce2^xx^	145.707 (6)
O10^i^—Li—O10	180.000 (1)	Mo2^ii^—Mo1—Ce2^xx^	90.187 (8)
O7^i^—Li—Mo2	138.20 (5)	Mo2—Mo1—Ce2^xx^	90.187 (8)
O7—Li—Mo2	41.80 (5)	O2—Mo1—Ce1^v^	57.59 (6)
O10^ii^—Li—Mo2	78.79 (4)	O2^ii^—Mo1—Ce1^v^	117.78 (5)
O10^iii^—Li—Mo2	101.21 (4)	O4^ii^—Mo1—Ce1^v^	111.62 (5)
O10^i^—Li—Mo2	136.78 (4)	O4—Mo1—Ce1^v^	53.03 (5)
O10—Li—Mo2	43.22 (4)	O1—Mo1—Ce1^v^	44.290 (8)
O7^i^—Li—Mo2^i^	41.80 (5)	Mo3^ii^—Mo1—Ce1^v^	144.943 (9)
O7—Li—Mo2^i^	138.20 (5)	Mo3—Mo1—Ce1^v^	96.635 (6)
O10^ii^—Li—Mo2^i^	101.21 (4)	Mo2^ii^—Mo1—Ce1^v^	151.707 (9)
O10^iii^—Li—Mo2^i^	78.79 (4)	Mo2—Mo1—Ce1^v^	99.651 (5)
O10^i^—Li—Mo2^i^	43.22 (4)	Ce2^xx^—Mo1—Ce1^v^	67.487 (5)
O10—Li—Mo2^i^	136.78 (4)	O2—Mo1—Ce1^ix^	117.78 (5)
Mo2—Li—Mo2^i^	180.000 (6)	O2^ii^—Mo1—Ce1^ix^	57.59 (6)
O7^i^—Li—Mo2^ii^	138.20 (5)	O4^ii^—Mo1—Ce1^ix^	53.03 (5)
O7—Li—Mo2^ii^	41.80 (5)	O4—Mo1—Ce1^ix^	111.62 (5)
O10^ii^—Li—Mo2^ii^	43.22 (4)	O1—Mo1—Ce1^ix^	44.290 (8)
O10^iii^—Li—Mo2^ii^	136.78 (4)	Mo3^ii^—Mo1—Ce1^ix^	96.635 (6)
O10^i^—Li—Mo2^ii^	101.21 (4)	Mo3—Mo1—Ce1^ix^	144.943 (9)
O10—Li—Mo2^ii^	78.79 (4)	Mo2^ii^—Mo1—Ce1^ix^	99.651 (5)
Mo2—Li—Mo2^ii^	54.260 (7)	Mo2—Mo1—Ce1^ix^	151.707 (9)
Mo2^i^—Li—Mo2^ii^	125.740 (7)	Ce2^xx^—Mo1—Ce1^ix^	67.487 (5)
O7^i^—Li—Mo2^iii^	41.80 (5)	Ce1^v^—Mo1—Ce1^ix^	87.964 (8)
O7—Li—Mo2^iii^	138.20 (5)	O3^xi^—Mo2—O2	86.18 (7)
O10^ii^—Li—Mo2^iii^	136.78 (4)	O3^xi^—Mo2—O7	85.95 (6)
O10^iii^—Li—Mo2^iii^	43.22 (4)	O2—Mo2—O7	87.51 (9)
O10^i^—Li—Mo2^iii^	78.79 (4)	O3^xi^—Mo2—O5	88.61 (7)
O10—Li—Mo2^iii^	101.21 (4)	O2—Mo2—O5	83.17 (7)
Mo2—Li—Mo2^iii^	125.740 (7)	O7—Mo2—O5	169.50 (9)
Mo2^i^—Li—Mo2^iii^	54.260 (7)	O3^xi^—Mo2—O10	87.58 (7)
Mo2^ii^—Li—Mo2^iii^	180.0	O2—Mo2—O10	172.63 (6)
O7^i^—Li—Ce1^xx^	128.23 (5)	O7—Mo2—O10	88.14 (9)
O7—Li—Ce1^xx^	51.77 (5)	O5—Mo2—O10	100.61 (7)
O10^ii^—Li—Ce1^xx^	135.89 (4)	O3^xi^—Mo2—Mo4^v^	92.95 (5)
O10^iii^—Li—Ce1^xx^	44.11 (4)	O2—Mo2—Mo4^v^	133.64 (5)
O10^i^—Li—Ce1^xx^	84.80 (4)	O7—Mo2—Mo4^v^	138.74 (8)
O10—Li—Ce1^xx^	95.20 (4)	O5—Mo2—Mo4^v^	50.48 (5)
Mo2—Li—Ce1^xx^	64.780 (4)	O10—Mo2—Mo4^v^	50.64 (4)
Mo2^i^—Li—Ce1^xx^	115.220 (4)	O3^xi^—Mo2—Mo1	132.54 (6)
Mo2^ii^—Li—Ce1^xx^	93.573 (4)	O2—Mo2—Mo1	46.41 (5)
Mo2^iii^—Li—Ce1^xx^	86.427 (4)	O7—Mo2—Mo1	89.31 (7)
O7^i^—Li—Ce1^xiv^	51.77 (5)	O5—Mo2—Mo1	87.76 (5)
O7—Li—Ce1^xiv^	128.23 (5)	O10—Mo2—Mo1	139.47 (5)
O10^ii^—Li—Ce1^xiv^	44.11 (4)	Mo4^v^—Mo2—Mo1	119.623 (9)
O10^iii^—Li—Ce1^xiv^	135.89 (4)	O3^xi^—Mo2—Mo5^xiv^	136.18 (6)
O10^i^—Li—Ce1^xiv^	95.20 (4)	O2—Mo2—Mo5^xiv^	137.57 (5)
O10—Li—Ce1^xiv^	84.80 (4)	O7—Mo2—Mo5^xiv^	92.57 (6)
Mo2—Li—Ce1^xiv^	115.220 (4)	O5—Mo2—Mo5^xiv^	97.57 (5)
Mo2^i^—Li—Ce1^xiv^	64.780 (4)	O10—Mo2—Mo5^xiv^	48.62 (4)
Mo2^ii^—Li—Ce1^xiv^	86.427 (4)	Mo4^v^—Mo2—Mo5^xiv^	60.718 (6)
Mo2^iii^—Li—Ce1^xiv^	93.573 (4)	Mo1—Mo2—Mo5^xiv^	91.153 (9)
Ce1^xx^—Li—Ce1^xiv^	180.000 (3)	O3^xi^—Mo2—Mo3	136.41 (5)
O3^iv^—Ce1—O12	122.15 (7)	O2—Mo2—Mo3	89.08 (4)
O3^iv^—Ce1—O1^v^	69.32 (7)	O7—Mo2—Mo3	137.13 (4)
O12—Ce1—O1^v^	134.79 (8)	O5—Mo2—Mo3	47.81 (4)
O3^iv^—Ce1—O8	120.66 (6)	O10—Mo2—Mo3	98.15 (4)
O12—Ce1—O8	116.24 (7)	Mo4^v^—Mo2—Mo3	60.674 (7)
O1^v^—Ce1—O8	77.76 (7)	Mo1—Mo2—Mo3	58.951 (7)
O3^iv^—Ce1—O10^vi^	82.31 (6)	Mo5^xiv^—Mo2—Mo3	62.917 (8)
O12—Ce1—O10^vi^	111.96 (7)	O3^xi^—Mo2—Mo2^ii^	133.86 (5)
O1^v^—Ce1—O10^vi^	112.96 (7)	O2—Mo2—Mo2^ii^	90.91 (5)
O8—Ce1—O10^vi^	66.91 (6)	O7—Mo2—Mo2^ii^	47.91 (4)
O3^iv^—Ce1—O4^v^	129.24 (6)	O5—Mo2—Mo2^ii^	136.76 (4)
O12—Ce1—O4^v^	80.26 (6)	O10—Mo2—Mo2^ii^	90.58 (5)
O1^v^—Ce1—O4^v^	64.64 (7)	Mo4^v^—Mo2—Mo2^ii^	120.433 (6)
O8—Ce1—O4^v^	68.88 (6)	Mo1—Mo2—Mo2^ii^	59.117 (5)
O10^vi^—Ce1—O4^v^	134.92 (5)	Mo5^xiv^—Mo2—Mo2^ii^	59.809 (5)
O3^iv^—Ce1—O2^v^	79.48 (6)	Mo3—Mo2—Mo2^ii^	89.470 (5)
O12—Ce1—O2^v^	78.39 (7)	O3^xi^—Mo2—Li	80.52 (5)
O1^v^—Ce1—O2^v^	59.97 (7)	O2—Mo2—Li	122.93 (5)
O8—Ce1—O2^v^	123.24 (5)	O7—Mo2—Li	36.66 (8)
O10^vi^—Ce1—O2^v^	161.79 (5)	O5—Mo2—Li	150.49 (5)
O4^v^—Ce1—O2^v^	59.85 (5)	O10—Mo2—Li	51.92 (4)
O3^iv^—Ce1—O11	140.28 (7)	Mo4^v^—Mo2—Li	102.456 (8)
O12—Ce1—O11	62.84 (7)	Mo1—Mo2—Li	119.677 (7)
O1^v^—Ce1—O11	139.21 (6)	Mo5^xiv^—Mo2—Li	72.991 (7)
O8—Ce1—O11	62.94 (5)	Mo3—Mo2—Li	135.603 (8)
O10^vi^—Ce1—O11	62.26 (6)	Mo2^ii^—Mo2—Li	62.870 (4)
O4^v^—Ce1—O11	90.07 (6)	O3^xi^—Mo2—Ce2^v^	43.12 (5)
O2^v^—Ce1—O11	134.84 (6)	O2—Mo2—Ce2^v^	59.43 (5)
O3^iv^—Ce1—O7^vii^	63.38 (5)	O7—Mo2—Ce2^v^	115.69 (5)
O12—Ce1—O7^vii^	69.19 (7)	O5—Mo2—Ce2^v^	55.05 (4)
O1^v^—Ce1—O7^vii^	131.52 (6)	O10—Mo2—Ce2^v^	117.60 (5)
O8—Ce1—O7^vii^	136.39 (7)	Mo4^v^—Mo2—Ce2^v^	89.410 (7)
O10^vi^—Ce1—O7^vii^	71.23 (7)	Mo1—Mo2—Ce2^v^	99.782 (8)
O4^v^—Ce1—O7^vii^	146.86 (6)	Mo5^xiv^—Mo2—Ce2^v^	149.571 (7)
O2^v^—Ce1—O7^vii^	100.38 (6)	Mo3—Mo2—Ce2^v^	98.774 (7)
O11—Ce1—O7^vii^	87.29 (5)	Mo2^ii^—Mo2—Ce2^v^	148.794 (4)
O3^iv^—Ce1—Mo1^v^	92.59 (5)	Li—Mo2—Ce2^v^	123.174 (6)
O12—Ce1—Mo1^v^	98.83 (5)	O8—Mo3—O5	86.67 (6)
O1^v^—Ce1—Mo1^v^	36.42 (6)	O8—Mo3—O11	85.73 (6)
O8—Ce1—Mo1^v^	88.19 (4)	O5—Mo3—O11	167.88 (8)
O10^vi^—Ce1—Mo1^v^	146.49 (4)	O8—Mo3—O4	89.16 (7)
O4^v^—Ce1—Mo1^v^	36.73 (4)	O5—Mo3—O4	84.24 (7)
O2^v^—Ce1—Mo1^v^	35.48 (3)	O11—Mo3—O4	86.22 (9)
O11—Ce1—Mo1^v^	126.78 (5)	O8—Mo3—Mo1	137.36 (5)
O7^vii^—Ce1—Mo1^v^	135.15 (5)	O5—Mo3—Mo1	88.70 (5)
O3^iv^—Ce1—Mo4^xv^	96.09 (5)	O11—Mo3—Mo1	90.50 (6)
O12—Ce1—Mo4^xv^	128.25 (6)	O4—Mo3—Mo1	48.20 (5)
O1^v^—Ce1—Mo4^xv^	88.36 (6)	O8—Mo3—Mo3^ii^	134.44 (5)
O8—Ce1—Mo4^xv^	34.03 (4)	O5—Mo3—Mo3^ii^	138.70 (4)
O10^vi^—Ce1—Mo4^xv^	34.97 (4)	O11—Mo3—Mo3^ii^	48.91 (4)
O4^v^—Ce1—Mo4^xv^	102.68 (4)	O4—Mo3—Mo3^ii^	91.45 (5)
O2^v^—Ce1—Mo4^xv^	147.69 (3)	Mo1—Mo3—Mo3^ii^	59.590 (5)
O11—Ce1—Mo4^xv^	65.48 (4)	O8—Mo3—Mo4^v^	91.12 (5)
O7^vii^—Ce1—Mo4^xv^	106.14 (6)	O5—Mo3—Mo4^v^	48.88 (5)
Mo1^v^—Ce1—Mo4^xv^	114.039 (6)	O11—Mo3—Mo4^v^	140.67 (7)
O3^iv^—Ce1—Mo2^vii^	31.40 (4)	O4—Mo3—Mo4^v^	132.99 (5)
O12—Ce1—Mo2^vii^	92.04 (5)	Mo1—Mo3—Mo4^v^	116.662 (9)
O1^v^—Ce1—Mo2^vii^	96.68 (5)	Mo3^ii^—Mo3—Mo4^v^	119.873 (6)
O8—Ce1—Mo2^vii^	145.48 (4)	O8—Mo3—Mo5	88.64 (5)
O10^vi^—Ce1—Mo2^vii^	84.91 (3)	O5—Mo3—Mo5	140.47 (5)
O4^v^—Ce1—Mo2^vii^	139.49 (4)	O11—Mo3—Mo5	48.74 (7)
O2^v^—Ce1—Mo2^vii^	79.64 (3)	O4—Mo3—Mo5	134.94 (5)
O11—Ce1—Mo2^vii^	121.66 (4)	Mo1—Mo3—Mo5	119.660 (8)
O7^vii^—Ce1—Mo2^vii^	34.91 (3)	Mo3^ii^—Mo3—Mo5	60.081 (5)
Mo1^v^—Ce1—Mo2^vii^	107.584 (6)	Mo4^v^—Mo3—Mo5	92.056 (9)
Mo4^xv^—Ce1—Mo2^vii^	112.839 (6)	O8—Mo3—Mo2	135.01 (5)
O12—Ce2—O3^ii^	133.58 (5)	O5—Mo3—Mo2	48.73 (4)
O12—Ce2—O3	133.58 (5)	O11—Mo3—Mo2	139.20 (4)
O3^ii^—Ce2—O3	79.20 (9)	O4—Mo3—Mo2	91.42 (4)
O12—Ce2—O1^vii^	149.28 (9)	Mo1—Mo3—Mo2	59.398 (8)
O3^ii^—Ce2—O1^vii^	65.37 (6)	Mo3^ii^—Mo3—Mo2	90.530 (5)
O3—Ce2—O1^vii^	65.37 (6)	Mo4^v^—Mo3—Mo2	57.266 (7)
O12—Ce2—O2^viii^	93.73 (7)	Mo5—Mo3—Mo2	120.425 (10)
O3^ii^—Ce2—O2^viii^	126.07 (5)	O8—Mo3—Mo4^xv^	46.92 (5)
O3—Ce2—O2^viii^	84.21 (6)	O5—Mo3—Mo4^xv^	90.84 (5)
O1^vii^—Ce2—O2^viii^	61.04 (6)	O11—Mo3—Mo4^xv^	90.85 (6)
O12—Ce2—O2^vii^	93.73 (7)	O4—Mo3—Mo4^xv^	136.07 (5)
O3^ii^—Ce2—O2^vii^	84.21 (6)	Mo1—Mo3—Mo4^xv^	175.622 (10)
O3—Ce2—O2^vii^	126.07 (5)	Mo3^ii^—Mo3—Mo4^xv^	118.702 (5)
O1^vii^—Ce2—O2^vii^	61.04 (6)	Mo4^v^—Mo3—Mo4^xv^	60.145 (7)
O2^viii^—Ce2—O2^vii^	65.50 (7)	Mo5—Mo3—Mo4^xv^	58.713 (6)
O12—Ce2—O5^v^	72.64 (6)	Mo2—Mo3—Mo4^xv^	117.351 (9)
O3^ii^—Ce2—O5^v^	66.93 (5)	O8—Mo3—Mo5^xiv^	133.82 (5)
O3—Ce2—O5^v^	104.01 (6)	O5—Mo3—Mo5^xiv^	94.57 (5)
O1^vii^—Ce2—O5^v^	132.27 (6)	O11—Mo3—Mo5^xiv^	97.51 (6)
O2^viii^—Ce2—O5^v^	166.30 (5)	O4—Mo3—Mo5^xiv^	136.95 (5)
O2^vii^—Ce2—O5^v^	115.69 (5)	Mo1—Mo3—Mo5^xiv^	88.790 (9)
O12—Ce2—O5^ix^	72.64 (6)	Mo3^ii^—Mo3—Mo5^xiv^	61.803 (5)
O3^ii^—Ce2—O5^ix^	104.01 (6)	Mo4^v^—Mo3—Mo5^xiv^	58.087 (6)
O3—Ce2—O5^ix^	66.93 (5)	Mo5—Mo3—Mo5^xiv^	61.970 (9)
O1^vii^—Ce2—O5^ix^	132.27 (6)	Mo2—Mo3—Mo5^xiv^	58.461 (7)
O2^viii^—Ce2—O5^ix^	115.69 (5)	Mo4^xv^—Mo3—Mo5^xiv^	86.906 (8)
O2^vii^—Ce2—O5^ix^	166.30 (5)	O8—Mo3—Ce3	42.95 (5)
O5^v^—Ce2—O5^ix^	59.64 (7)	O5—Mo3—Ce3	55.13 (5)
O12—Ce2—O2^ix^	77.39 (4)	O11—Mo3—Ce3	113.38 (5)
O3^ii^—Ce2—O2^ix^	140.99 (6)	O4—Mo3—Ce3	60.69 (5)
O3—Ce2—O2^ix^	62.17 (6)	Mo1—Mo3—Ce3	102.416 (8)
O1^vii^—Ce2—O2^ix^	100.42 (3)	Mo3^ii^—Mo3—Ce3	149.964 (5)
O2^viii^—Ce2—O2^ix^	58.34 (6)	Mo4^v^—Mo3—Ce3	89.212 (7)
O2^vii^—Ce2—O2^ix^	122.06 (3)	Mo5—Mo3—Ce3	131.587 (7)
O5^v^—Ce2—O2^ix^	115.54 (5)	Mo2—Mo3—Ce3	100.481 (7)
O5^ix^—Ce2—O2^ix^	57.35 (5)	Mo4^xv^—Mo3—Ce3	80.844 (7)
O12—Ce2—O2^v^	77.39 (4)	Mo5^xiv^—Mo3—Ce3	146.743 (8)
O3^ii^—Ce2—O2^v^	62.17 (6)	O6—Mo4—O8^xvi^	85.31 (9)
O3—Ce2—O2^v^	140.99 (6)	O6—Mo4—O5^v^	89.00 (9)
O1^vii^—Ce2—O2^v^	100.42 (3)	O8^xvi^—Mo4—O5^v^	173.10 (7)
O2^viii^—Ce2—O2^v^	122.06 (3)	O6—Mo4—O10^v^	91.20 (7)
O2^vii^—Ce2—O2^v^	58.34 (6)	O8^xvi^—Mo4—O10^v^	82.35 (7)
O5^v^—Ce2—O2^v^	57.35 (5)	O5^v^—Mo4—O10^v^	101.70 (7)
O5^ix^—Ce2—O2^v^	115.54 (5)	O6—Mo4—O9	79.29 (5)
O2^ix^—Ce2—O2^v^	154.73 (7)	O8^xvi^—Mo4—O9	87.84 (5)
O12—Ce2—Mo1^vii^	111.35 (6)	O5^v^—Mo4—O9	87.27 (4)
O3^ii^—Ce2—Mo1^vii^	94.00 (4)	O10^v^—Mo4—O9	166.89 (5)
O3—Ce2—Mo1^vii^	94.00 (4)	O6—Mo4—Mo2^v^	96.21 (6)
O1^vii^—Ce2—Mo1^vii^	37.93 (6)	O8^xvi^—Mo4—Mo2^v^	133.42 (5)
O2^viii^—Ce2—Mo1^vii^	36.42 (4)	O5^v^—Mo4—Mo2^v^	51.11 (4)
O2^vii^—Ce2—Mo1^vii^	36.42 (4)	O10^v^—Mo4—Mo2^v^	51.11 (5)
O5^v^—Ce2—Mo1^vii^	150.15 (3)	O9—Mo4—Mo2^v^	138.338 (10)
O5^ix^—Ce2—Mo1^vii^	150.15 (3)	O6—Mo4—Mo5^xvii^	140.45 (5)
O2^ix^—Ce2—Mo1^vii^	93.84 (3)	O8^xvi^—Mo4—Mo5^xvii^	87.85 (5)
O2^v^—Ce2—Mo1^vii^	93.84 (3)	O5^v^—Mo4—Mo5^xvii^	99.03 (5)
O12—Ce2—Mo2^v^	98.05 (3)	O10^v^—Mo4—Mo5^xvii^	49.26 (5)
O3^ii^—Ce2—Mo2^v^	35.57 (4)	O9—Mo4—Mo5^xvii^	139.345 (9)
O3—Ce2—Mo2^v^	106.07 (5)	Mo2^v^—Mo4—Mo5^xvii^	61.952 (8)
O1^vii^—Ce2—Mo2^v^	97.73 (3)	O6—Mo4—Mo3^v^	137.41 (8)
O2^viii^—Ce2—Mo2^v^	150.68 (4)	O8^xvi^—Mo4—Mo3^v^	136.74 (4)
O2^vii^—Ce2—Mo2^v^	86.93 (4)	O5^v^—Mo4—Mo3^v^	48.54 (5)
O5^v^—Ce2—Mo2^v^	37.81 (3)	O10^v^—Mo4—Mo3^v^	99.64 (5)
O5^ix^—Ce2—Mo2^v^	93.48 (4)	O9—Mo4—Mo3^v^	93.467 (8)
O2^ix^—Ce2—Mo2^v^	150.67 (3)	Mo2^v^—Mo4—Mo3^v^	62.060 (7)
O2^v^—Ce2—Mo2^v^	37.12 (4)	Mo5^xvii^—Mo4—Mo3^v^	63.801 (8)
Mo1^vii^—Ce2—Mo2^v^	114.434 (4)	O6—Mo4—Mo4^xviii^	127.74 (5)
O12^v^—Ce3—O8	132.16 (5)	O8^xvi^—Mo4—Mo4^xviii^	88.58 (4)
O12^v^—Ce3—O8^x^	132.16 (5)	O5^v^—Mo4—Mo4^xviii^	91.83 (4)
O8—Ce3—O8^x^	80.35 (8)	O10^v^—Mo4—Mo4^xviii^	139.18 (4)
O12^v^—Ce3—O4^v^	108.37 (7)	O9—Mo4—Mo4^xviii^	48.606 (5)
O8—Ce3—O4^v^	72.90 (6)	Mo2^v^—Mo4—Mo4^xviii^	123.722 (10)
O8^x^—Ce3—O4^v^	115.46 (5)	Mo5^xvii^—Mo4—Mo4^xviii^	90.884 (6)
O12^v^—Ce3—O4^xi^	108.37 (7)	Mo3^v^—Mo4—Mo4^xviii^	61.730 (7)
O8—Ce3—O4^xi^	115.46 (5)	O6—Mo4—Mo3^xvi^	130.47 (8)
O8^x^—Ce3—O4^xi^	72.90 (6)	O8^xvi^—Mo4—Mo3^xvi^	45.63 (4)
O4^v^—Ce3—O4^xi^	68.25 (8)	O5^v^—Mo4—Mo3^xvi^	139.29 (4)
O12^v^—Ce3—O5^x^	72.46 (7)	O10^v^—Mo4—Mo3^xvi^	88.72 (5)
O8—Ce3—O5^x^	103.13 (6)	O9—Mo4—Mo3^xvi^	90.635 (7)
O8^x^—Ce3—O5^x^	65.41 (5)	Mo2^v^—Mo4—Mo3^xvi^	120.793 (9)
O4^v^—Ce3—O5^x^	175.45 (6)	Mo5^xvii^—Mo4—Mo3^xvi^	58.843 (8)
O4^xi^—Ce3—O5^x^	115.94 (5)	Mo3^v^—Mo4—Mo3^xvi^	91.111 (9)
O12^v^—Ce3—O5	72.47 (7)	Mo4^xviii^—Mo4—Mo3^xvi^	58.125 (7)
O8—Ce3—O5	65.41 (5)	O6—Mo4—Mo4^ii^	38.07 (5)
O8^x^—Ce3—O5	103.13 (6)	O8^xvi^—Mo4—Mo4^ii^	88.38 (5)
O4^v^—Ce3—O5	115.94 (5)	O5^v^—Mo4—Mo4^ii^	84.74 (5)
O4^xi^—Ce3—O5	175.45 (6)	O10^v^—Mo4—Mo4^ii^	129.14 (5)
O5^x^—Ce3—O5	59.82 (7)	O9—Mo4—Mo4^ii^	41.395 (5)
O12^v^—Ce3—O4^x^	74.97 (3)	Mo2^v^—Mo4—Mo4^ii^	120.433 (6)
O8—Ce3—O4^x^	143.69 (6)	Mo5^xvii^—Mo4—Mo4^ii^	176.099 (7)
O8^x^—Ce3—O4^x^	63.90 (5)	Mo3^v^—Mo4—Mo4^ii^	119.873 (6)
O4^v^—Ce3—O4^x^	127.22 (4)	Mo4^xviii^—Mo4—Mo4^ii^	90.0
O4^xi^—Ce3—O4^x^	61.42 (6)	Mo3^xvi^—Mo4—Mo4^ii^	118.702 (5)
O5^x^—Ce3—O4^x^	57.32 (5)	O6—Mo4—Ce1^xvi^	75.70 (7)
O5—Ce3—O4^x^	115.02 (5)	O8^xvi^—Mo4—Ce1^xvi^	40.64 (4)
O12^v^—Ce3—O4	74.97 (3)	O5^v^—Mo4—Ce1^xvi^	141.03 (4)
O8—Ce3—O4	63.90 (5)	O10^v^—Mo4—Ce1^xvi^	44.48 (5)
O8^x^—Ce3—O4	143.69 (6)	O9—Mo4—Ce1^xvi^	123.412 (8)
O4^v^—Ce3—O4	61.42 (6)	Mo2^v^—Mo4—Ce1^xvi^	94.531 (7)
O4^xi^—Ce3—O4	127.22 (3)	Mo5^xvii^—Mo4—Ce1^xvi^	73.927 (8)
O5^x^—Ce3—O4	115.02 (5)	Mo3^v^—Mo4—Ce1^xvi^	137.453 (8)
O5—Ce3—O4	57.32 (5)	Mo4^xviii^—Mo4—Ce1^xvi^	125.945 (10)
O4^x^—Ce3—O4	149.80 (6)	Mo3^xvi^—Mo4—Ce1^xvi^	70.101 (6)
O12^v^—Ce3—O11^v^	59.67 (8)	Mo4^ii^—Mo4—Ce1^xvi^	102.511 (4)
O8—Ce3—O11^v^	130.85 (5)	O11—Mo5—O10^xiv^	85.23 (7)
O8^x^—Ce3—O11^v^	130.85 (5)	O11—Mo5—O10^vi^	85.23 (7)
O4^v^—Ce3—O11^v^	59.89 (5)	O10^xiv^—Mo5—O10^vi^	84.89 (9)
O4^xi^—Ce3—O11^v^	59.89 (5)	O11—Mo5—Mo4^xix^	93.800 (8)
O5^x^—Ce3—O11^v^	123.33 (5)	O10^xiv^—Mo5—Mo4^xix^	48.96 (4)
O5—Ce3—O11^v^	123.33 (5)	O10^vi^—Mo5—Mo4^xix^	133.64 (5)
O4^x^—Ce3—O11^v^	81.27 (4)	O11—Mo5—Mo4^xv^	93.800 (8)
O4—Ce3—O11^v^	81.27 (4)	O10^xiv^—Mo5—Mo4^xv^	133.64 (5)
O12^v^—Ce3—O1^v^	157.12 (8)	O10^vi^—Mo5—Mo4^xv^	48.96 (4)
O8—Ce3—O1^v^	61.54 (5)	Mo4^xix^—Mo5—Mo4^xv^	172.199 (15)
O8^x^—Ce3—O1^v^	61.54 (5)	O11—Mo5—Mo3	48.79 (4)
O4^v^—Ce3—O1^v^	53.99 (5)	O10^xiv^—Mo5—Mo3	134.00 (5)
O4^xi^—Ce3—O1^v^	53.99 (5)	O10^vi^—Mo5—Mo3	91.23 (5)
O5^x^—Ce3—O1^v^	126.41 (5)	Mo4^xix^—Mo5—Mo3	122.183 (10)
O5—Ce3—O1^v^	126.41 (5)	Mo4^xv^—Mo5—Mo3	62.444 (6)
O4^x^—Ce3—O1^v^	103.27 (3)	O11—Mo5—Mo3^ii^	48.79 (4)
O4—Ce3—O1^v^	103.27 (3)	O10^xiv^—Mo5—Mo3^ii^	91.23 (5)
O11^v^—Ce3—O1^v^	97.45 (6)	O10^vi^—Mo5—Mo3^ii^	134.00 (5)
O12^v^—Ce3—Mo3	96.37 (3)	Mo4^xix^—Mo5—Mo3^ii^	62.444 (6)
O8—Ce3—Mo3	35.79 (4)	Mo4^xv^—Mo5—Mo3^ii^	122.183 (10)
O8^x^—Ce3—Mo3	107.81 (4)	Mo3—Mo5—Mo3^ii^	59.838 (9)
O4^v^—Ce3—Mo3	81.55 (4)	O11—Mo5—Mo2^xiv^	133.96 (4)
O4^xi^—Ce3—Mo3	145.52 (4)	O10^xiv^—Mo5—Mo2^xiv^	48.76 (5)
O5^x^—Ce3—Mo3	93.92 (4)	O10^vi^—Mo5—Mo2^xiv^	91.12 (5)
O5—Ce3—Mo3	37.35 (3)	Mo4^xix^—Mo5—Mo2^xiv^	57.330 (6)
O4^x^—Ce3—Mo3	151.21 (4)	Mo4^xv^—Mo5—Mo2^xiv^	117.621 (10)
O4—Ce3—Mo3	37.32 (4)	Mo3—Mo5—Mo2^xiv^	176.566 (12)
O11^v^—Ce3—Mo3	118.510 (8)	Mo3^ii^—Mo5—Mo2^xiv^	119.773 (6)
O1^v^—Ce3—Mo3	95.02 (2)	O11—Mo5—Mo2^vi^	133.96 (4)
O12^v^—Ce3—Mo3^x^	96.37 (3)	O10^xiv^—Mo5—Mo2^vi^	91.12 (5)
O8—Ce3—Mo3^x^	107.81 (4)	O10^vi^—Mo5—Mo2^vi^	48.76 (5)
O8^x^—Ce3—Mo3^x^	35.79 (4)	Mo4^xix^—Mo5—Mo2^vi^	117.621 (10)
O4^v^—Ce3—Mo3^x^	145.52 (4)	Mo4^xv^—Mo5—Mo2^vi^	57.330 (6)
O4^xi^—Ce3—Mo3^x^	81.55 (4)	Mo3—Mo5—Mo2^vi^	119.773 (6)
O5^x^—Ce3—Mo3^x^	37.35 (3)	Mo3^ii^—Mo5—Mo2^vi^	176.566 (12)
O5—Ce3—Mo3^x^	93.92 (4)	Mo2^xiv^—Mo5—Mo2^vi^	60.381 (9)
O4^x^—Ce3—Mo3^x^	37.32 (4)	O11—Mo5—Mo3^xiv^	138.63 (4)
O4—Ce3—Mo3^x^	151.21 (4)	O10^xiv^—Mo5—Mo3^xiv^	94.72 (5)
O11^v^—Ce3—Mo3^x^	118.510 (8)	O10^vi^—Mo5—Mo3^xiv^	136.07 (5)
O1^v^—Ce3—Mo3^x^	95.02 (2)	Mo4^xix^—Mo5—Mo3^xiv^	58.113 (6)
Mo3—Ce3—Mo3^x^	119.929 (9)	Mo4^xv^—Mo5—Mo3^xiv^	114.493 (10)
O12^v^—Ce3—Mo1^v^	122.20 (7)	Mo3—Mo5—Mo3^xiv^	118.030 (9)
O8—Ce3—Mo1^v^	87.20 (4)	Mo3^ii^—Mo5—Mo3^xiv^	89.919 (9)
O8^x^—Ce3—Mo1^v^	87.20 (4)	Mo2^xiv^—Mo5—Mo3^xiv^	58.622 (7)
O4^v^—Ce3—Mo1^v^	35.35 (4)	Mo2^vi^—Mo5—Mo3^xiv^	87.395 (10)
O4^xi^—Ce3—Mo1^v^	35.35 (4)	O11—Mo5—Mo3^vi^	138.63 (4)
O5^x^—Ce3—Mo1^v^	148.03 (3)	O10^xiv^—Mo5—Mo3^vi^	136.07 (5)
O5—Ce3—Mo1^v^	148.03 (3)	O10^vi^—Mo5—Mo3^vi^	94.72 (5)
O4^x^—Ce3—Mo1^v^	96.76 (3)	Mo4^xix^—Mo5—Mo3^vi^	114.493 (10)
O4—Ce3—Mo1^v^	96.76 (3)	Mo4^xv^—Mo5—Mo3^vi^	58.113 (6)
O11^v^—Ce3—Mo1^v^	62.53 (4)	Mo3—Mo5—Mo3^vi^	89.919 (9)
O1^v^—Ce3—Mo1^v^	34.92 (5)	Mo3^ii^—Mo5—Mo3^vi^	118.030 (9)
Mo3—Ce3—Mo1^v^	110.725 (4)	Mo2^xiv^—Mo5—Mo3^vi^	87.395 (10)
Mo3^x^—Ce3—Mo1^v^	110.725 (4)	Mo2^vi^—Mo5—Mo3^vi^	58.622 (7)
O12^v^—Ce3—O9^xxv^	113.66 (7)	Mo3^xiv^—Mo5—Mo3^vi^	56.395 (9)
O8—Ce3—O9^xxv^	52.86 (4)	O11—Mo5—Mo5^xiv^	97.23 (6)
O8^x^—Ce3—O9^xxv^	52.86 (4)	O10^xiv^—Mo5—Mo5^xiv^	137.55 (4)
O4^v^—Ce3—O9^xxv^	125.08 (4)	O10^vi^—Mo5—Mo5^xiv^	137.55 (4)
O4^xi^—Ce3—O9^xxv^	125.08 (4)	Mo4^xix^—Mo5—Mo5^xiv^	88.648 (6)
O5^x^—Ce3—O9^xxv^	51.42 (4)	Mo4^xv^—Mo5—Mo5^xiv^	88.648 (6)
O5—Ce3—O9^xxv^	51.42 (4)	Mo3—Mo5—Mo5^xiv^	61.593 (9)
O4^x^—Ce3—O9^xxv^	97.25 (4)	Mo3^ii^—Mo5—Mo5^xiv^	61.593 (9)
O4—Ce3—O9^xxv^	97.25 (4)	Mo2^xiv^—Mo5—Mo5^xiv^	115.053 (13)
O11^v^—Ce3—O9^xxv^	173.33 (4)	Mo2^vi^—Mo5—Mo5^xiv^	115.053 (13)
O1^v^—Ce3—O9^xxv^	89.22 (5)	Mo3^xiv^—Mo5—Mo5^xiv^	56.437 (9)
Mo3—Ce3—O9^xxv^	60.555 (5)	Mo3^vi^—Mo5—Mo5^xiv^	56.437 (9)
Mo3^x^—Ce3—O9^xxv^	60.555 (5)	Mo1—O1—Ce1^v^	99.29 (6)
Mo1^v^—Ce3—O9^xxv^	124.139 (7)	Mo1—O1—Ce1^ix^	99.29 (6)
O12^v^—Ce3—Ce1^xi^	36.60 (3)	Ce1^v^—O1—Ce1^ix^	157.89 (13)
O8—Ce3—Ce1^xi^	168.71 (4)	Mo1—O1—Ce2^xx^	90.06 (9)
O8^x^—Ce3—Ce1^xi^	108.76 (4)	Ce1^v^—O1—Ce2^xx^	95.93 (6)
O4^v^—Ce3—Ce1^xi^	107.70 (4)	Ce1^ix^—O1—Ce2^xx^	95.93 (6)
O4^xi^—Ce3—Ce1^xi^	74.40 (4)	Mo1—O1—Ce3^v^	76.55 (7)
O5^x^—Ce3—Ce1^xi^	75.73 (4)	Ce1^v^—O1—Ce3^v^	86.38 (6)
O5—Ce3—Ce1^xi^	105.23 (3)	Ce1^ix^—O1—Ce3^v^	86.38 (6)
O4^x^—Ce3—Ce1^xi^	44.87 (3)	Ce2^xx^—O1—Ce3^v^	166.61 (11)
O4—Ce3—Ce1^xi^	106.11 (3)	Mo1—O2—Mo2	84.01 (7)
O11^v^—Ce3—Ce1^xi^	47.87 (3)	Mo1—O2—Ce2^xx^	91.22 (7)
O1^v^—Ce3—Ce1^xi^	128.32 (4)	Mo2—O2—Ce2^xx^	131.36 (8)
Mo3—Ce3—Ce1^xi^	132.947 (5)	Mo1—O2—Ce1^v^	86.94 (6)
Mo3^x^—Ce3—Ce1^xi^	78.291 (4)	Mo2—O2—Ce1^v^	142.24 (7)
Mo1^v^—Ce3—Ce1^xi^	99.649 (6)	Ce2^xx^—O2—Ce1^v^	85.31 (6)
O9^xxv^—Ce3—Ce1^xi^	127.147 (5)	Mo1—O2—Ce2^v^	144.38 (7)
O6^xii^—Ce4—O6	180.0	Mo2—O2—Ce2^v^	83.45 (6)
O6^xii^—Ce4—O3	101.93 (7)	Ce2^xx^—O2—Ce2^v^	121.66 (6)
O6—Ce4—O3	78.07 (7)	Ce1^v^—O2—Ce2^v^	82.93 (5)
O6^xii^—Ce4—O3^xii^	78.07 (7)	Mo2^ix^—O3—Ce1^xxi^	111.27 (8)
O6—Ce4—O3^xii^	101.93 (7)	Mo2^ix^—O3—Ce2	101.31 (7)
O3—Ce4—O3^xii^	180.00 (6)	Ce1^xxi^—O3—Ce2	103.98 (7)
O6^xii^—Ce4—O3^ii^	101.93 (7)	Mo2^ix^—O3—Ce4	127.21 (8)
O6—Ce4—O3^ii^	78.07 (7)	Ce1^xxi^—O3—Ce4	108.51 (6)
O3—Ce4—O3^ii^	77.58 (9)	Ce2—O3—Ce4	101.22 (6)
O3^xii^—Ce4—O3^ii^	102.42 (9)	Mo1—O4—Mo3	81.91 (7)
O6^xii^—Ce4—O3^xiii^	78.07 (7)	Mo1—O4—Ce3^v^	98.59 (7)
O6—Ce4—O3^xiii^	101.93 (7)	Mo3—O4—Ce3^v^	116.62 (7)
O3—Ce4—O3^xiii^	102.42 (9)	Mo1—O4—Ce1^v^	90.24 (6)
O3^xii^—Ce4—O3^xiii^	77.58 (9)	Mo3—O4—Ce1^v^	144.66 (8)
O3^ii^—Ce4—O3^xiii^	180.00 (6)	Ce3^v^—O4—Ce1^v^	98.58 (6)
O6^xii^—Ce4—Ce2	109.59 (7)	Mo1—O4—Ce3	142.82 (8)
O6—Ce4—Ce2	70.41 (7)	Mo3—O4—Ce3	81.99 (6)
O3—Ce4—Ce2	38.98 (4)	Ce3^v^—O4—Ce3	118.58 (6)
O3^xii^—Ce4—Ce2	141.02 (4)	Ce1^v^—O4—Ce3	83.95 (5)
O3^ii^—Ce4—Ce2	38.98 (4)	Mo3—O5—Mo4^v^	82.58 (6)
O3^xiii^—Ce4—Ce2	141.02 (4)	Mo3—O5—Mo2	83.46 (6)
O6^xii^—Ce4—Ce2^xii^	70.41 (7)	Mo4^v^—O5—Mo2	78.42 (6)
O6—Ce4—Ce2^xii^	109.59 (7)	Mo3—O5—Ce3	87.52 (6)
O3—Ce4—Ce2^xii^	141.02 (4)	Mo4^v^—O5—Ce3	125.09 (7)
O3^xii^—Ce4—Ce2^xii^	38.98 (4)	Mo2—O5—Ce3	153.48 (9)
O3^ii^—Ce4—Ce2^xii^	141.02 (4)	Mo3—O5—Ce2^v^	150.69 (9)
O3^xiii^—Ce4—Ce2^xii^	38.98 (4)	Mo4^v^—O5—Ce2^v^	122.56 (7)
Ce2—Ce4—Ce2^xii^	180.000 (1)	Mo2—O5—Ce2^v^	87.13 (5)
O6^xii^—Ce4—Ce1^xvi^	113.22 (5)	Ce3—O5—Ce2^v^	88.70 (5)
O6—Ce4—Ce1^xvi^	66.78 (5)	Mo4—O6—Mo4^ii^	103.86 (10)
O3—Ce4—Ce1^xvi^	144.81 (4)	Mo4—O6—Ce4	127.46 (5)
O3^xii^—Ce4—Ce1^xvi^	35.19 (4)	Mo4^ii^—O6—Ce4	127.46 (5)
O3^ii^—Ce4—Ce1^xvi^	93.18 (4)	Li—O7—Mo2^ii^	101.54 (10)
O3^xiii^—Ce4—Ce1^xvi^	86.82 (4)	Li—O7—Mo2	101.54 (10)
Ce2—Ce4—Ce1^xvi^	121.134 (3)	Mo2^ii^—O7—Mo2	84.19 (8)
Ce2^xii^—Ce4—Ce1^xvi^	58.866 (3)	Li—O7—Ce1^xx^	97.91 (7)
O6^xii^—Ce4—Ce1^xxi^	66.78 (5)	Mo2^ii^—O7—Ce1^xx^	160.55 (13)
O6—Ce4—Ce1^xxi^	113.22 (5)	Mo2—O7—Ce1^xx^	92.16 (3)
O3—Ce4—Ce1^xxi^	35.19 (4)	Li—O7—Ce1^xxii^	97.91 (7)
O3^xii^—Ce4—Ce1^xxi^	144.81 (4)	Mo2^ii^—O7—Ce1^xxii^	92.16 (3)
O3^ii^—Ce4—Ce1^xxi^	86.82 (4)	Mo2—O7—Ce1^xxii^	160.55 (13)
O3^xiii^—Ce4—Ce1^xxi^	93.18 (4)	Ce1^xx^—O7—Ce1^xxii^	84.94 (6)
Ce2—Ce4—Ce1^xxi^	58.866 (3)	Mo3—O8—Mo4^xv^	87.45 (7)
Ce2^xii^—Ce4—Ce1^xxi^	121.134 (3)	Mo3—O8—Ce3	101.26 (6)
Ce1^xvi^—Ce4—Ce1^xxi^	180.000 (3)	Mo4^xv^—O8—Ce3	132.81 (8)
O6^xii^—Ce4—Ce1^xxiv^	113.22 (5)	Mo3—O8—Ce1	113.93 (8)
O6—Ce4—Ce1^xxiv^	66.78 (5)	Mo4^xv^—O8—Ce1	105.34 (6)
O3—Ce4—Ce1^xxiv^	93.18 (4)	Ce3—O8—Ce1	112.60 (7)
O3^xii^—Ce4—Ce1^xxiv^	86.82 (4)	Mo4^ii^—O9—Mo4^xviii^	180.000 (11)
O3^ii^—Ce4—Ce1^xxiv^	144.81 (4)	Mo4^ii^—O9—Mo4	97.211 (10)
O3^xiii^—Ce4—Ce1^xxiv^	35.19 (4)	Mo4^xviii^—O9—Mo4	82.789 (10)
Ce2—Ce4—Ce1^xxiv^	121.134 (3)	Mo4^ii^—O9—Mo4^xxiii^	82.789 (10)
Ce2^xii^—Ce4—Ce1^xxiv^	58.866 (3)	Mo4^xviii^—O9—Mo4^xxiii^	97.211 (10)
Ce1^xvi^—Ce4—Ce1^xxiv^	74.878 (4)	Mo4—O9—Mo4^xxiii^	180.000 (11)
Ce1^xxi^—Ce4—Ce1^xxiv^	105.122 (4)	Mo4^ii^—O9—Ce3^v^	94.201 (6)
O6^xii^—Ce4—Ce1^xxvi^	66.78 (5)	Mo4^xviii^—O9—Ce3^v^	85.799 (6)
O6—Ce4—Ce1^xxvi^	113.22 (5)	Mo4—O9—Ce3^v^	94.201 (6)
O3—Ce4—Ce1^xxvi^	86.82 (4)	Mo4^xxiii^—O9—Ce3^v^	85.799 (6)
O3^xii^—Ce4—Ce1^xxvi^	93.18 (4)	Mo4^ii^—O9—Ce3^xxiv^	85.799 (6)
O3^ii^—Ce4—Ce1^xxvi^	35.19 (4)	Mo4^xviii^—O9—Ce3^xxiv^	94.201 (6)
O3^xiii^—Ce4—Ce1^xxvi^	144.81 (4)	Mo4—O9—Ce3^xxiv^	85.799 (6)
Ce2—Ce4—Ce1^xxvi^	58.866 (3)	Mo4^xxiii^—O9—Ce3^xxiv^	94.201 (6)
Ce2^xii^—Ce4—Ce1^xxvi^	121.134 (3)	Ce3^v^—O9—Ce3^xxiv^	180.000 (5)
Ce1^xvi^—Ce4—Ce1^xxvi^	105.122 (4)	Mo4^v^—O10—Mo5^xiv^	81.78 (6)
Ce1^xxi^—Ce4—Ce1^xxvi^	74.878 (4)	Mo4^v^—O10—Mo2	78.26 (6)
Ce1^xxiv^—Ce4—Ce1^xxvi^	180.000 (3)	Mo5^xiv^—O10—Mo2	82.62 (6)
O2—Mo1—O2^ii^	92.35 (10)	Mo4^v^—O10—Li	162.57 (9)
O2—Mo1—O4^ii^	167.83 (8)	Mo5^xiv^—O10—Li	100.44 (7)
O2^ii^—Mo1—O4^ii^	88.28 (7)	Mo2—O10—Li	84.86 (6)
O2—Mo1—O4	88.28 (7)	Mo4^v^—O10—Ce1^vi^	100.54 (7)
O2^ii^—Mo1—O4	167.83 (8)	Mo5^xiv^—O10—Ce1^vi^	111.85 (7)
O4^ii^—Mo1—O4	88.58 (10)	Mo2—O10—Ce1^vi^	165.29 (8)
O2—Mo1—O1	83.18 (7)	Li—O10—Ce1^vi^	94.68 (6)
O2^ii^—Mo1—O1	83.18 (7)	Mo5—O11—Mo3	82.47 (8)
O4^ii^—Mo1—O1	84.83 (7)	Mo5—O11—Mo3^ii^	82.47 (8)
O4—Mo1—O1	84.83 (7)	Mo3—O11—Mo3^ii^	82.19 (8)
O2—Mo1—Mo3^ii^	142.09 (6)	Mo5—O11—Ce1^ii^	100.65 (7)
O2^ii^—Mo1—Mo3^ii^	93.36 (5)	Mo3—O11—Ce1^ii^	176.28 (11)
O4^ii^—Mo1—Mo3^ii^	49.89 (5)	Mo3^ii^—O11—Ce1^ii^	96.162 (13)
O4—Mo1—Mo3^ii^	93.59 (5)	Mo5—O11—Ce1	100.65 (7)
O1—Mo1—Mo3^ii^	134.71 (5)	Mo3—O11—Ce1	96.162 (13)
O2—Mo1—Mo3	93.36 (5)	Mo3^ii^—O11—Ce1	176.28 (11)
O2^ii^—Mo1—Mo3	142.09 (6)	Ce1^ii^—O11—Ce1	85.28 (6)
O4^ii^—Mo1—Mo3	93.59 (5)	Mo5—O11—Ce3^v^	179.63 (10)
O4—Mo1—Mo3	49.89 (5)	Mo3—O11—Ce3^v^	97.25 (8)
O1—Mo1—Mo3	134.71 (5)	Mo3^ii^—O11—Ce3^v^	97.25 (8)
Mo3^ii^—Mo1—Mo3	60.821 (10)	Ce1^ii^—O11—Ce3^v^	79.62 (6)
O2—Mo1—Mo2^ii^	95.29 (6)	Ce1—O11—Ce3^v^	79.62 (6)
O2^ii^—Mo1—Mo2^ii^	49.58 (6)	Ce2—O12—Ce3^v^	113.01 (11)
O4^ii^—Mo1—Mo2^ii^	94.36 (5)	Ce2—O12—Ce1^ii^	109.67 (6)
O4—Mo1—Mo2^ii^	142.46 (6)	Ce3^v^—O12—Ce1^ii^	107.36 (6)
O1—Mo1—Mo2^ii^	132.72 (5)	Ce2—O12—Ce1	109.67 (6)
Mo3^ii^—Mo1—Mo2^ii^	61.651 (7)	Ce3^v^—O12—Ce1	107.36 (6)
Mo3—Mo1—Mo2^ii^	92.568 (10)	Ce1^ii^—O12—Ce1	109.69 (11)
O2—Mo1—Mo2	49.58 (6)		

Symmetry codes: (i) −*x*−1, −*y*−1, −*z*+2; (ii) *x*, −*y*−1, *z*; (iii) −*x*−1, *y*, −*z*+2; (iv) −*x*−1/2, *y*+1/2, −*z*; (v) −*x*−1/2, −*y*−1/2, −*z*+1; (vi) −*x*−1, *y*, −*z*+1; (vii) *x*, *y*, *z*−1; (viii) *x*, −*y*−1, *z*−1; (ix) −*x*−1/2, *y*−1/2, −*z*+1; (x) *x*, −*y*, *z*; (xi) −*x*−1/2, *y*+1/2, −*z*+1; (xii) −*x*, −*y*−1, −*z*; (xiii) −*x*, *y*, −*z*; (xiv) −*x*−1, −*y*−1, −*z*+1; (xv) *x*−1/2, −*y*−1/2, *z*; (xvi) *x*+1/2, −*y*−1/2, *z*; (xvii) *x*+1/2, *y*+1/2, *z*; (xviii) −*x*, *y*, −*z*+1; (xix) *x*−1/2, *y*−1/2, *z*; (xx) *x*, *y*, *z*+1; (xxi) −*x*−1/2, *y*−1/2, −*z*; (xxii) *x*, −*y*−1, *z*+1; (xxiii) −*x*, −*y*−1, −*z*+1; (xxiv) *x*+1/2, *y*−1/2, *z*; (xxv) *x*−1/2, *y*+1/2, *z*; (xxvi) −*x*−1/2, −*y*−1/2, −*z*.
